# Reporting completeness of scoping reviews in orthodontic literature up to 2022. An empirical study

**DOI:** 10.1093/ejo/cjad022

**Published:** 2023-05-15

**Authors:** Filippos Mikelis, Despina Koletsi

**Affiliations:** School of Dentistry, National and Kapodistrian University of Athens, Athens, Greece; Clinic of Orthodontics and Pediatric Dentistry, Center of Dental Medicine, University of Zurich, Zurich, Switzerland; Meta-Research Innovation Center at Stanford (METRICS), Stanford University, California, USA

## Abstract

**Aim:**

To assess the quality of reporting of Scoping Reviews (ScRs) in Orthodontics according to the PRISMA Extension Checklist for Scoping Reviews (PRISMA-ScR). Our secondary aim was to identify publication characteristics, such as year of publication, journal, inclusion of a reporting guideline, and study registration, associated with ScRs reporting quality.

**Materials and Methods:**

Pubmed, Scopus, and Web of Science Core Collection were searched as of 1 August 2022 for identification of orthodontic ScRs. This was supplemented by electronic searches within the contents of eleven specialty journals. The item-specific and overall reporting quality score of the examined orthodontic ScRs, based on the PRISMA Extension Checklist for Scoping Reviews were recorded. Association of reporting quality score with publication characteristics was further examined.

**Results:**

A total of 40 ScRs were identified and included, with a mean reporting quality score of 73.0 per cent (standard deviation = 14). The majority of studies were published from 2020 onwards (32/40; 80.0%). Of the most adequately reported items were the summary of the evidence description in the Discussion (38/40; 95.0%) and the selection of the sources of evidence in the Results section (34/40; 85.0%). Protocol registration and reporting of limitations were missed in almost half of the ScRs (19/40; 47.5%), while less than half studies were adequately justified (18/40; 45.0%). According to the multivariable linear regression, adherence to appropriate reporting guidelines resulted in improved reporting quality score by 10 per cent (β-coefficient: 0.10; 95% CI: 0.002, 0.19; *P* = 0.04), conditional on year and journal of publication. Year, journal of publication, and registration practices did not appear as significant predictors (*P* > 0.05 in all instances).

**Conclusions:**

The reporting quality of the examined orthodontic ScRs was suboptimal, with questionable justification for their conduct and certain items being mostly affected.

## Introduction

Reporting quality and certainty of the evidence have been of utmost importance in contemporary orthodontic research (1,2). The need to provide clinicians and patients with evidence-based decisions in a way that is the most advantageous and the least harmful, has been governing scientific output of authors and researchers; as such, available evidence is thoroughly scrutinized and subsequently utilized. It is nowadays widely acknowledged that Systematic Reviews (SRs) and meta-analyses are at the summit of the evidence pyramid (3). The SRs’ aims are to gather knowledge and explore a strictly delineated subject using ‘explicit, systematic methods to collate and synthesize findings of studies that address a clearly formulated question’ (4). The PRISMA (Preferred Reporting Items for Systematic Reviews and Meta-Analyses) reporting guidelines have been established and updated to the latest version to include a variety of key reporting domains, comprising 27 reporting items (5). A clear and transparent reporting may allow the readership first to assess the rigorousness of the methodologies applied and followed, second, to interpret the findings in a solid manner and third, to evaluate the reproducibility and usability of the Review in healthcare decision making (4).

In 2009, Grant and Booth described a special review type which focussed on a ‘preliminary assessment of potential size and scope of available research literature’ which ‘aims to identify the nature and extent of research evidence’ (6). This was framed under the term Scoping Review and was further delineated as an investigation of evidence over a wider topic of interest, having no special focus or any strictly defined research question (7). Lately, review publications reported as Scoping Reviews (ScRs) have emerged in Dentistry and Orthodontics, as well. Early empirical research suggested that the authors of the ScRs used generally inadequate and non-standardized reporting methods (8). Thus, if evidence is poorly justified and presented, and data are not adequately supported and evidence-based, articles’ validity and integrity are doubtful.

A recently published report related to oral health ScRs reveals that the latter are not adequately justified, methodologically sound, and/or have registered protocols (9). These early findings from oral health research were confirmed by a subsequent follow-up about rationale justification practices of Orthodontic ScRs (10). However, the level of reporting, registration, and reproducibility strategies framed under the existing reporting guidelines for ScRs have not been studied in the orthodontic literature.

Therefore, the primary aim of the present empirical study was to identify and record the completeness of reporting of Scoping Reviews in Orthodontics according to the PRISMA Extension guidelines and Checklist for Scoping Reviews (PRISMA-ScR). As a secondary aim, we examined the association of ScRs’ reporting with publication characteristics such as year of publication, journal, inclusion of a reporting guideline, and study registration.

## Methods

### Eligibility criteria

The detailed methodology for study eligibility and inclusion is presented in our previous publication in the field (10). In essence, studies were selected based on the terminology used, indicating the conduct of a ‘scoping review’, in the title, abstract, or material and methods section of the manuscript.

### Search strategy

The following databases were searched until 1 August 2022, for eligible studies: MEDLINE via PubMed, Scopus, Web of Science (core collection). No filter, time, or other restriction was used. The full search strategy is presented in Supplementary material. Keywords such as ‘scoping review’ and ‘orthodontic’ were used. We also scrutinized the electronic contents of the entirety of indexed scientific journals in the field. These were: the American Journal of Orthodontics and Dentofacial Orthopaedics (AJODO), European Journal of Orthodontics (EJO), Orthodontics and Craniofacial Research (OCR), Angle Orthodontist (ANGLE), Progress in Orthodontics, International Orthodontics, Journal of the World Federation of Orthodontists, Turkish Journal of Orthodontics, APOS Trends in Orthodontics, Seminars in Orthodontics, Korean Journal of Orthodontics.

### Data extraction/recording and calibration

Data extraction was conducted independently by two authors (FM and DK), and the final standardized form was the product of consensus agreement between the two reviewers, after settlement of any disagreement.

A similar consensus-based methodology was followed for the detailed recording of adherence to the PRISMA Extension Checklist for Scoping Reviews (PRISMA-ScR), reported by Tricco *et al*. (8). An initial calibration phase was conducted in 10 articles and the unweighted kappa statistic was used per item to assess agreement.

### Development of the checklist/scoring and primary outcome

The PRISMA-ScR checklist was used to assess the quality of reporting over a score of 20 items. The range of score per item was 0 to 1, including 0.5. Ranking as zero (0) pertained to no description, 0.5 to inadequate description, and 1 to adequate description. If an item comprised several sub-categories, to achieve the score of ‘1—adequate’, a clear reporting of all sub-categories was anticipated. If one or more, but not all sub-items were described, then a rating of ‘0.5—inadequate’ was recorded. The primary outcome comprised the overall percentage score. A score equal to 20 corresponded to a 100 per cent. Additional two items were also separately recorded, but since these were considered optional items in the PRISMA-ScR checklist, they did not contribute to the overall percentage score; both items were related to the methodology and results of the critical appraisal of individual, included sources of evidence.

### Exploration of potential predictors of the primary outcome

A number of publication characteristics were also recorded: the year of publication, the type of journal (either specialty or not), the continent of authorship based on the institutional affiliation of the corresponding author, the number of authors that co-authored the publication, whether the authors of the ScRs reported having followed or adhered to any specific relevant reporting guidelines and whether the ScR was registered or not.

### Statistical analysis

Descriptive statistics were performed for the overall percentage reporting score according to PRISMA ScR, across the levels of the aforementioned publication characteristics. Frequency distribution of the scores per item of the PRISMA ScRs checklist was also undertaken.

Univariable and multivariable linear regression was performed, with beta-coefficients and respective 95 per cent Confidence Intervals (CIs) for the effect of year, journal, appropriateness of reporting guidelines used and registration, on the percentage reporting PRISMA ScR score.

The predictors were inserted sequentially one at a time in the initial model (forward stepwise variable selection) and best-fit model selection was based on the information criteria Akaike Information Criterion (AIC), and Bayesian Information Criterion (BIC). The model which minimized the information criteria was selected. The unweighted kappa statistic was used to assess inter-rater agreement per item at the initial piloting phase. The predefined level of significance was set at *P* < 0.05 (two-sided). All analyses were conducted with Stata version 15.1 (Stata Corporation, College Station, Texas, USA).

## Results

A total of 215 unique reports of studies were identified by the unified search strategy and 40 Scoping Reviews were considered eligible for inclusion in the present evaluation of their reporting quality. Within the sample, 80 per cent had been published since 2020 with similar numbers being published in orthodontic (18/40; 45.0%) and non-specialty journals (22/40; 55.0%) (Table 1). The corresponding author affiliation of the ScRs corresponded primarily to European Institutions (17/40; 42.5%) and in almost half ScRs the article was co-authored by four to five reviewers (18/40; 45.0%). Twenty-one ScRs followed established and appropriate guidelines to report their methodology and findings (52.5%), while the majority were not registered (34/40; 85.0%).

**Table 1.** Descriptive statistics for the percentage reporting score according to PRISMA ScR (*n* = 40)

	Reporting score			
	*N*	%	Mean [median]	SD [IQR]
Year				
2016	1	2.5	[63]	–
2017	2	5.0	[65]	[25]
2018	1	2.5	[57]	–
2019	4	10.0	[69]	[7]
2020	7	17.5	72	14
2021	17	42.5	74	15
2022–	8	20.0	81	14
Journal				
Orthodontic	18	45.0	76	14
Non-specialty	22	55.0	71	14
Continent				
America	7	17.5	79	13
Europe	17	42.5	70	16
Asia/other	16	40.0	75	12
No. authors				
1–3	13	32.5	68	13
4–5	18	45.0	78	13
≥6	9	22.5	72	16
Appropriate reporting guidelines (if any)				
No	19	47.5	67	14
Yes	21	52.5	79	11
Registration				
No	34	85.0	73	13
Yes	6	15.0	75	20
Overall	40	100	73	14

SD, standard deviation; IQR, interquartile range.

Mean [median] and SD [IQR] are reported as percentage scores.

The overall reporting quality score was 73 per cent (standard deviation: 14). The interrater agreement kappa values for the individual items of the checklist ranged between 0.68 (95% CI: 0.48–0.84) and 0.84 (95% CI: 0.80–1.00), denoting at least substantial agreement. The overall percentage score across different levels of the examined variables is presented in Table 1. Evidence presentation and description in the Discussion (38/40; 95.0%), selection of the sources of evidence in the Results section (34/40; 85.0%) as well as the Title (37/40; 92.5%) were the items most frequently adequately reported and the sole three items that received a rating of ‘1—adequately reported’ in more than 80 per cent of the sample.

**Figure 1:**
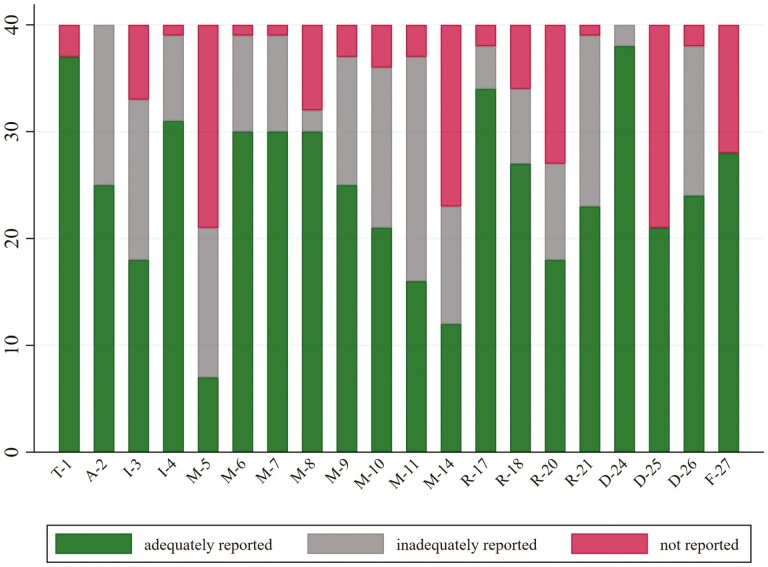
Bar-chart of frequency distribution of non-reported, inadequately reported, and adequately reported items according to the PRISMA ScR checklist.

The most prevalent items that were completely missed by the authors of the ScRs, and rated as ‘0—no description’ were: reporting of protocol development and registration in the Methodology section (19/40; 47.5%), reporting of limitations in the Discussion (19/40; 47.5%), reporting of methods of handling and summarizing the data in the Methodology (17/40; 42.5%) and reporting of the results of individual sources of evidence according to the ScR question and objectives (13/40; 32.5%) (Table 2; Figure 1). In addition, regarding the two optional items reported in the PRISMA-ScRs guidelines, namely the critical appraisal of the individual sources of evidence both in the Methodology and the Results section, only three studies (3/40; 7.5%) presented an adequate description throughout; in one further study, although the authors claimed an intention to critically appraise the evidence they identified, there was no further reporting of their findings in this respect.

**Table 2.** Frequency distribution of scores per item of the PRISMA ScR checklist

PRISMA ScR item	No description		Inadequate		Adequate		Total (100%)
	*N*	%	*N*	%	*N*	%	*N*
1. Title	3	7.5	0	0.0	37	92.5	40
2. Abstract	0	0.0	15	37.5	25	62.5	40
3. Introduction—Rationale	7	17.5	15	37.5	18	45.0	40
4. Introduction—Objectives	1	2.5	8	20.0	31	77.5	40
5. Methods—Protocol and registration	19	47.5	14	35.0	7	17.5	40
6. Methods—Eligibility criteria	1	2.5	9	22.5	30	75.0	40
7. Methods—Information sources	1	2.5	9	22.5	30	75.0	40
8. Methods—Search	8	20.0	2	5.0	30	75.0	40
9. Methods—Selection of sources of evidence	3	7.5	12	30.0	25	62.5	40
10. Methods—Data charting process	4	10.0	15	37.5	21	52.5	40
11. Methods-Data items	3	7.5	21	52.5	16	40.0	40
14. Methods—Synthesis of results	17	42.5	11	27.5	12	30.0	40
17. Results—Selection of sources of evidence	2	5.0	4	10.0	34	85.0	40
18. Results—Characteristics of sources of evidence	6	15.0	7	17.5	27	67.5	40
20. Results—Results of individual sources of evidence	13	32.5	9	22.5	18	45.0	40
21. Results—Synthesis of results	1	2.5	16	40.0	23	57.5	40
24. Discussion—Summary of evidence	0	0.0	2	5.0	38	95.0	40
25. Discussion—Limitations	19	47.5	0	0.0	21	52.5	40
26. Discussion—Conclusions	2	5.0	14	35.0	24	60.0	40
27. Funding	12	30.0	0	0.0	28	70.0	40

There was evidence that adherence to appropriate reporting guidelines upon the development of the publishable report of the ScR, resulted in improved reporting quality score by 10 per cent (β-coefficient: 0.10; 95% CI: 0.002, 0.19; *P* = 0.04), conditional on year and journal of publication, according to the multivariable linear regression. Contrary, neither year of publication, nor journal were found to have a significant effect on reporting score (*P* > 0.05 in both instances), based on the adjusted model (Table 3).

**Table 3.** Univariable and multivariable linear regression with beta- coefficients and respective Confidence Intervals (CIs) for the effect of year, journal, appropriateness of reporting guidelines used and registration, on the percentage reporting PRISMA ScR score

Category	Univariable			Multivariable^*^		
	β-coefficient	95% CI	*P*-value	β-coefficient	95% CI	*P*-value
Year			0.05			0.32
Per unit	0.03	0.00, 0.06		0.02	–0.02, 0.05	
Journal			0.26			0.36
Non-specialty	Reference					
Orthodontic	0.05	–0.04, 0.14		0.04	–0.05, 0.12	
Appropriate reporting guidelines			0.004			0.04
No	Reference			Reference		
Yes	0.12	0.04, 0.20		0.10	0.002, 0.19	
Registration			0.81			
No	Reference					
Yes	0.01	–0.11, 0.14				

^*^The presented model indicated the lowest values of both AIC and BIC information criteria.

## Discussion

### Summary of findings

The overall findings of the present empirical study give a representative picture of the reporting quality of the published ScRs in Orthodontics to date. Following prior research on justification of ScRs (9,10), the present study confirmed a suboptimal level of presentation and reporting for these types of Reviews. Variability across reporting quality items was detected, with some key aspects reported even in less than half of the examined articles.

### Prior research

No direct comparison can be made with previous similar meta-epidemiological reports on ScRs, either in Orthodontics, or in Dentistry overall or within the medical literature, since this is the first study attempting to map the current methodological practices in terms of quality and level of reporting of ScRs. The timing of the design and conduct of the present study was considered crucial, since we identified a clear increase in publication in Scoping Reviews in Orthodontics, over the very last years, as it was also confirmed by our search strategy results, over a wide spectrum of journals and databases. As such, our goal was to highlight the limitations of the existing evidence stemming from ScRs, in order to frame current practices, propose methods of improvement, and further deter research waste (11).

Likewise, lack of transparent methodological and reporting practices in systematic reviews in Orthodontics has already been confirmed by prior research. Issues of publication bias (12), heterogeneity perspectives (13), use of quality assessment tools (14) or clear reporting of the certainty of the evidence provided by the SRs are some examples (2). Similarly, the quality of reporting in Orthodontic SRs has been examined during the last decade. Early evidence, in 2013, has demonstrated a 64 per cent overall percentage score of adherence to the PRISMA reporting guidelines (15), while the reporting levels have also been associated to the methodological quality of the SRs (15,16). Most severely impacted domains were registration, publication bias reporting, reporting of summary measures, and planned analyses (15). Indeed, a variety of methodological and reporting flaws in these domains have been convulsing relevant research in Oral Health and specialties other than Orthodontics as well (17–19).

### Findings in context

The introduction of Scoping Reviews in Orthodontics has been rather recent and it might well be possible that authors of ScRs are not well educated or familiar with the rationale behind such types of evidence syntheses. In essence, 80 per cent of the sample was published from 2020 onwards. It might also be argued that authors could probably confuse the rationale behind a scoping review methodology with that of a systematic review or even that of a narrative review. One of the ‘key elements’ of a systematic review of the literature is to map the level of quality and risk of bias of the included studies, irrespective of the inclusion of a meta-analysis. This is commonly not applicable or not implemented in a scoping review, thus ScRs might be considered by authors easier and faster to undertake and publish, with minimal advising and consultation with expert methodologists in the field; following this, one might speculate that ScRs do not guarantee identification and mapping of solid evidence to fill in the knowledge gaps and promote research and decision making perspectives. If this is additionally considered in conjunction with the identified lack of justification in most of the ScRs examined in this study, as also confirmed by previous studies (9,10), their contribution in the evidence base is yet to be recognized and confirmed.

Of the most severely impacted domains in terms of completeness of reporting was protocol conduct and registration for the ScRs. Even in 14 of 40 studies, where the existence of a protocol was reported, this did not appear registered, while in half of the studies there was no information about either existence of a protocol or any registration. It is known that registration practices have been identified as a primary indicator for reproducible and transparent research (20–24).

Furthermore, adequate presentation of the methodology of synthesis and summarizing of data was followed only in a third of the sample examined. It has been reported that clear and transparent description of the methodology followed in a published article is key to enable reproduction and validation of research and research practices (25,26). This was also the case for SR methodology followed in articles published a decade ago, in Orthodontics (16), while there is no recently updated report in the field. A detailed methodological description of the framework of an intended research plan would apparently reduce the risk for *post hoc* attempted modifications and changes in methods and plans by the researchers. In essence, the description of the methodology should be in complete agreement with what is reported in the evidence synthesis in the results section of a systematic or a scoping review. It was also noteworthy that only half of the assessed articles included a limitations section, streamlining any speculations for deficiencies in the scoping review process or generalizability of the results and potential implications and effects of non-adherence to reporting such shortcomings. Contrary, evidence from similar studies in SRs in the field indicate adequate description of limitations of SRs, denoting very good adherence to reporting guidelines in this respect (16). Such differences may be attributed to the limited knowledge of ScR methodology by the authors overall, misinterpretation of the objectives and value of these studies, and a potential failure to acknowledge key elements of reporting of research.

We identified adherence to reporting guidelines as the sole factor positively associated with reporting quality and standards. Early efforts to guide the reporting of ‘scoping studies’ in biomedicine were documented back in 2005 (27), and further recorded in a more consistent and precise manner in 2015, an effort framed by members of the Joanna Briggs Institute (28). Likewise, the PRISMA statement developed an extension for the guidance in reporting ScRs (8), thus reflecting the differences in the methodological and conceptual framework governing these types of reviews (29). Consistent strategies to improve adherence to reporting guidelines have already led to increased quality of reporting in other types of studies in orthodontic literature (30).

### Strengths and limitations

Overall, the present study constitutes the first attempt to critically appraise and map the quality of reporting and contextual information derived from ScRs in the field. It builds upon a previously conducted report on the justification and rationale behind such studies (10). This has come at the early phase of their appearance in the orthodontic literature and it follows that authors, reviewers, and editors are expected to be additionally alert regarding the rational and clear methodological strategies followed upon conduct, drafting, and dissemination of the findings of these studies. One might assume that the sample of this study might be limited, however, it forms a rather complete picture of the available evidence in the field so far. Our search was conducted in three major databases and supplemented by separate electronic searches within eleven orthodontic journals, almost up to date. A further extent in our search until the end of 2022 to identify additional studies yielded only three additional studies; this would not justify any re-analysis of our sample upon inclusion of the latter, since it was not considered adequate to impact on the existing findings.

## Conclusions

Our findings suggest non-optimal reporting of orthodontic scoping reviews. Awareness should be raised in authors, reviewers, and editors, on concrete examination of the rational and methodological rigorousness of scoping reviews, in relation to the research question these types of studies intend to answer. Thereafter, adherence to reporting guidelines should be enhanced and facilitated.

## Supplementary Material

cjad022_suppl_Supplementary_Materials

## Data Availability

The data underlying this article will be shared on reasonable request to the corresponding author.
