# Supplementary material for: Reporting completeness of scoping reviews in orthodontic literature up to 2022. An empirical study
Source: Eur J Orthod. 2023 May 15;45(4):444–9. doi: 10.1093/ejo/cjad022 (PMC10411490; doi:10.1093/ejo/cjad022)
Supplement: cjad022_suppl_Supplementary_Materials [file cjad022_suppl_supplementary_materials.docx]

**Supplementary Material.** Search Strategy for study selection, for MEDLINE (via PubMed) and adapted for the other databases.

Date: August 1^st^, 2022

All fields

No filters, or language/ time restriction

((orthodontic) OR (tooth movement) OR (root resorption) OR (maxillary expansion) OR (fixed orthodontic appliances) OR (orthodontic aligners) OR (tooth agenesis) OR (Class I malocclusion) OR (Class II malocclusion) OR (Class III malocclusion) OR (mandibular advancement) OR (maxillary protraction)) AND ((scoping review) OR (mapping review))
